# Aberrant TERT expression: linking chronic inflammation to hepatocellular carcinoma^†^

**DOI:** 10.1002/path.6421

**Published:** 2025-04-11

**Authors:** Rui Dong, Gregoire Najjar, Cagatay Günes, André Lechel

**Affiliations:** ^1^ Department of Internal Medicine I University Hospital Ulm Ulm Germany; ^2^ Department of Urology University Hospital Ulm Ulm Germany

**Keywords:** TERT, NF‐κB promoter, p21 ubiquitination, p53 absence, HCC, hepatocarcinogenesis

## Abstract

Telomerase reverse transcriptase (TERT), the catalytic enzyme component of telomerase, plays multiple roles in cellular biology. Its canonical function is primarily associated with telomere maintenance and genomic stability. In addition, several studies revealed critical non‐canonical extra‐telomeric functions of TERT in various cellular processes, including cell proliferation and survival, DNA damage response, transcription, signal transduction, and metabolic regulation, both in normal and in cancer cells. Notably, TERT is aberrantly upregulated in more than 80% of hepatocellular carcinoma (HCC) cases, making it an important target in liver cancer research. However, due to the diversity and complexity of TERT's functions *in vivo*, the precise mechanisms by which TERT contributes to the initiation and progression of HCC remain unclear. A recent study published in *The Journal of Pathology* using the *Alb‐Cre;Tert*Tg mouse model and clinical HCC samples addresses the role of TERT in hepatocarcinogenesis. The study demonstrates that TERT promotes cell cycle progression and hepatocarcinogenesis by enhancing NF‐κB promoter activity and facilitating the ubiquitination of p21. Notably, absence of functional p53 accelerates liver tumor development in TERT transgenic mice. These findings further underscore the critical role of TERT in inflammation‐driven hepatocarcinogenesis and provide new insights into its underlying mechanisms. © 2025 The Author(s). *The Journal of Pathology* published by John Wiley & Sons Ltd on behalf of The Pathological Society of Great Britain and Ireland.

Hepatocellular carcinoma (HCC) is the most common type of primary liver cancer, manifesting predominantly in the context of chronic liver disease. Chronic liver disease is marked by the acceleration of hepatocyte turnover, a consequence of prolonged inflammation and the accumulation of reactive oxygen species (ROS). This process is accompanied by progressive telomere shortening. Telomeres, crucial protective structures at the ends of chromosomes, are paramount for maintaining genomic stability. Telomere shortening leads to chromosomal instability, which subsequently becomes a key driver of tumorigenesis [1]. The maintenance of telomere functionality primarily depends on telomerase activity, which is regulated by its catalytic subunit, telomerase reverse transcriptase (TERT). In the context of normal physiological conditions, TERT expression is repressed in differentiated hepatocytes [2]. Indeed, TERT is downregulated during early embryogenesis in most human tissues. Only proliferating B‐ and T‐cells as well as stem and progenitor cells express telomerase in adult human tissues. Consequently, TERT repression is believed to be a pivotal tumor‐protective mechanism. In fact, reactivation of telomerase has been observed in the vast majority of human cancer cells. In hepatocytes, TERT reactivation occurs due to an altered E2F/Rb pathway or by mutations in the *TERT* promoter during the early stages of HCC. In addition, chromosomal translocation and hepatitis B virus (HBV) integration into the *TERT* gene locus are frequently observed in HCC samples. This results in TERT upregulation and reactivation of telomerase, thereby driving tumor initiation and progression [2]. In the classical view, telomerase reactivation is required for telomere maintenance, thereby delaying telomere shortening and sustaining cell division [3].

In recent years, multiple reports have provided substantial evidence that the telomerase complex or the TERT subunit alone plays critical roles beyond mere telomere elongation. Indeed, these non‐canonical functions of TERT/telomerase also influence various biological processes, including the regulation of inflammatory responses, inhibition of apoptosis, and modulation of the cell cycle [3]. For instance, TERT may act as a transcriptional co‐regulator, promoting cell proliferation by regulating the expression of cyclins and influencing cell cycle progression, particularly the G1/S phase transition [2]. TERT also regulates a plethora of key signaling pathways, namely Wnt/β‐catenin and NF‐κB, as well as metabolic genes involved in glycolysis and oxidative phosphorylation to meet the energy demands of cancer cells, while interacting with the PI3K/Akt pathway to further enhance cell survival and proliferation [4, 5]. Of critical importance, TERT/telomerase suppresses the activation of DNA damage response (DDR) pathways and allows the survival of pre‐malignant cells, and promotes malignant transformation by inhibiting senescence and apoptotic pathways [3]. In summary, these findings suggest that TERT upregulation is a core driver of tumorigenesis, including HCC development and progression. However, the specific roles and underlying mechanisms of TERT in the progression from chronic liver disease to HCC remain to be elucidated.

In a recent issue of *The Journal of Pathology*, Mishima *et al* established a liver‐specific TERT overexpression mouse model (*Alb‐Cre;Tert*Tg), providing an important tool for precisely investigating the specific functions of TERT *in vivo* [6]. The authors made comparisons between telomere lengths in the livers of *Alb‐Cre;Tert*Tg mice and control mice under conditions of chronic inflammation. Paradoxically, despite an anticipated telomere length increase, the telomeres of *Tert*‐overexpressing mice exhibited accelerated shortening under these conditions, in comparison to both control mice and transgenic mice lacking inflammatory signals.

The study successfully recapitulated the early genetic alterations commonly observed in HCC by combining *Tert* overexpression, chronic inflammation, and *Trp53* deletion. This combination of factors highlights the critical roles of *TERT* and *TP53* in liver carcinogenesis and progression. Furthermore, the authors demonstrate that the synergy between *Tert* overexpression and *Trp53* loss significantly accelerates the development of liver tumors [6]. The results are consistent with previous investigations, which demonstrated the synergistic effect of telomerase expression and *Trp53* deficiency on liver tumor progression [7]. This observation underscores the crucial role of TERT's non‐canonical functions in the progression of chronic hepatitis and tumorigenesis, extending beyond its classical role in telomere maintenance.

Based on gene set enrichment analysis, this study further delves into the role of TERT in the activation of NF‐κB signaling and cell cycle regulation. Further analysis *in vivo* and *in vitro* shed light on direct protein binding of TERT and p65, implying that this interaction activates promoters of downstream pro‐inflammatory genes, in the absence of external stimuli [6]. These findings establish TERT as a key promoter in inflammation‐driven hepatocarcinogenesis. Furthermore, the authors show a clear interruption of the cell cycle by *Tert* overexpression *in vivo*, or conversely by *TERT* silencing *in vitro*. This observation was explained by the modulation of different cyclin proteins. Mechanistically, the authors provide evidence that TERT interacts with p21 (CDKN1A), resulting in the ubiquitination and proteosomal degradation of p21. This process alleviates the inhibitory effect of p21 on CDK2, and promotes progression from the G1 to the S phase, thereby enhancing cellular proliferation. Additionally, the negative correlation between TERT and p21 observed in human HCC samples further corroborates the *in vitro* and *in vivo* findings, underscoring the clinical relevance of this regulatory pathway [6]. These results provide compelling evidence for the pivotal role of TERT in driving inflammatory responses and promoting cell cycle progression (Figure 1).

**Figure 1:**
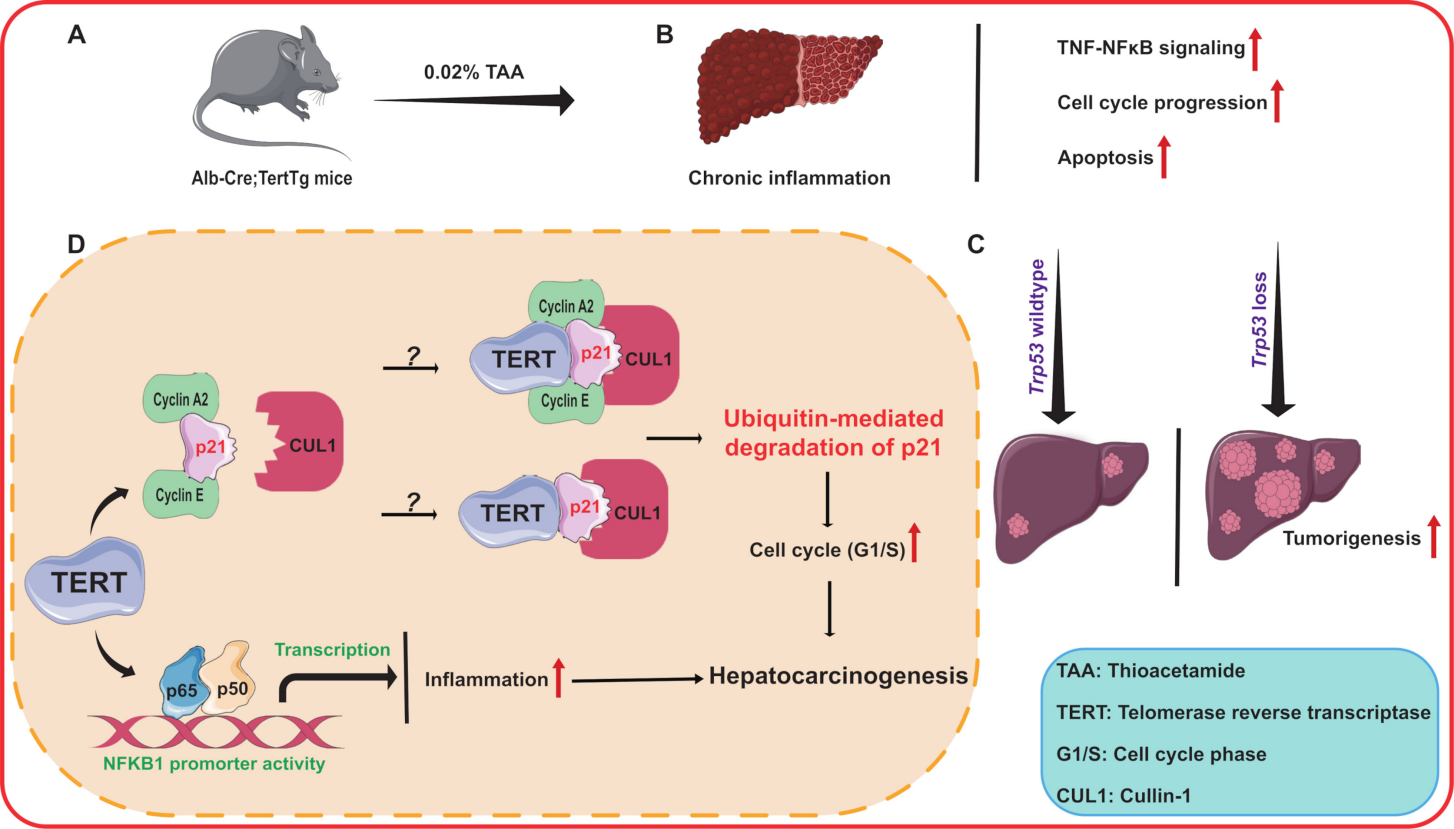
The role of TERT in inflammation‐associated hepatocarcinogenesis. (A) In a liver‐specific TERT overexpression mouse model (*Alb‐Cre;Tert*Tg), chronic liver inflammation was induced using thioacetamide (TAA), creating a platform to study the interplay between TERT and inflammatory carcinogenesis. (B) TERT overexpression alone does not significantly increase liver tumor incidence but enhances the expression of gene sets implicated in inflammatory carcinogenesis, highlighting its role in modulating the inflammatory microenvironment. (C) The absence of p53 synergizes with TERT overexpression to accelerate liver tumorigenesis, emphasizing the cooperative effects of these alterations in promoting malignant transformation. (D) Mechanistically, TERT interacts with the NF‐κB p65 subunit to enhance *NF‐κB1* promoter activity, upregulating pro‐inflammatory gene expression. Additionally, TERT binds to p21, cyclin A2, and cyclin E during G1, facilitating ubiquitin‐mediated degradation of p21 via cullin‐1. This degradation relieves p21‐mediated inhibition of cyclin‐dependent kinase (CDK) activity, thereby promoting cell cycle progression and contributing to hepatocarcinogenesis. The exact mechanisms by which TERT binds to p21 and interacts with cyclin A2 and cyclin E remain to be elucidated. The artwork used in this figure contains modified images from Servier Medical Art (https://smart.servier.com/). Servier Medical Art by Servier is licensed under a Creative Commons Attribution 4.0 Unported License (https://creativecommons.org/licenses/by/4.0/).

During the progression of chronic hepatitis, the aberrant upregulation of TERT may promote carcinogenesis through various mechanisms. However, transgenic mice revealed that telomerase knockout results in the shortening of telomeres in hepatocytes, which is associated with accelerated liver cirrhosis [8]. Conversely, telomerase therapy has been shown to mitigate this process [8]. This phenomenon may suggest that telomerase activation could have dual roles in genome stability and tumor formation. As cells divide, telomeres gradually shorten, which limits the proliferative capacity of cells to some extent. The degradation of telomeres triggers cellular senescence or apoptosis, thereby suppressing tumorigenesis. However, aberrant repair or elongation of telomeres can lead to chromosomal rearrangements, deletions, or amplifications, increasing the risk of tumor development. Therefore, overexpression of telomerase is closely related to the mechanisms underlying cancer pathogenesis. Over 80% of HCCs exhibit telomerase activity, allowing these cells to bypass the limitations imposed during normal cell proliferation [9]. The present study demonstrated that liver‐specific overexpression of *Tert* in the context of chronic inflammation resulted in a substantial decrease in telomere length. This finding indicates that, under these specific conditions, the function of TERT may transition from its conventional role in telomere maintenance to a novel pathway involving NF‐κB activation and atypical modulation of the cell cycle [6]. However, it remains unclear whether TERT interacts with inflammatory signaling molecules (such as NF‐κB) to increase the expression of pro‐inflammatory genes, thereby exacerbating the inflammatory response, or whether it accelerates the degradation of p21, promoting cell division and senescence, thereby weakening the protective effect of telomerase on telomeres.

Nevertheless, the present study demonstrates that aberrant upregulation of TERT promotes carcinogenesis in the progression of chronic hepatitis and liver cancer by enhancing inflammatory responses and regulating cell proliferation. As a limitation, the interconnection between TERT and p53 in cell cycle control makes it hard to distinguish the specific role of TERT in p21 modulation. Moreover, despite the similarity of telomere sequences between humans and mice, in inbred laboratory mice, *Tert*/telomerase is expressed constitutively in the majority of cells, including hepatocytes. The length of telomeres in these mice is approximately five times longer than human telomeres, which suggests substantial differences between humans and mice. In humans, telomere shortening plays a major role in genome instability and genetic crisis during tumorigenesis [10].

In summary, Mishima *et al* [6] present findings that underscore the significance of TERT reactivation during periods of chronic liver inflammation. TERT, as evidenced by the research, facilitates proliferation through NF‐κB activation and p21 degradation. However, this process renders cells susceptible to additional tumor‐initiating mutations, thereby highlighting a complex interplay between cellular processes and the development of cancer. In future research, utilization of patient‐derived samples, organoid models, or xenograft models is recommended for further validation. The incorporation of these models will ensure the broader applicability of the results obtained.

## Author contributions statement

RD, GN, CG and AL wrote the manuscript. All authors read and approved the final manuscript.

## Data Availability

Data sharing is not applicable to this article as no new data were created or analyzed in this study.
